# The federal government commissioner for patient issues in Germany: initial analysis of the user inquiries

**DOI:** 10.1186/1472-6963-7-24

**Published:** 2007-02-22

**Authors:** Nils Schneider, Marie-Luise Dierks, Gabriele Seidel, Friedrich W Schwartz

**Affiliations:** 1 Department of Epidemiology, Social Medicine and Health System Research, Hannover Medical School, Carl-Neuberg-Str. 1, D-30625 Hannover, Germany

## Abstract

**Background:**

The political objective in many countries worldwide is to give better consideration to the interests of patients within the health system. The establishment of a federal government commissioner for the issues of patients in the health system in Germany in 2004 is part of these endeavours. The structure and field of activities of this institution has been unique so far. This study investigates for the first time the inquiries the commissioner receives from the public.

**Methods:**

A 33% sampling (n = 850) of the written inquiries (correspondence and e-mails) addressed to the commissioner in the first six months of the year 2005 (n = 2580) was investigated. In a procedure comprising combined qualitative and quantitative levels, the material was thematically encoded and the inquiries allocated to the resulting categories (multiple nominations). The results are presented in descriptive form and investigated especially with respect to sex and age-specific differences. The interdependences between the categorized criteria are analysed.

**Results:**

The inquirers are equally spread out amongst the sexes (49% women, 51% men). Older persons outweigh the younger (69% over 60 years). In most cases the issues take the form of claims (72%, n = 609). In every fifth inquiry (n = 168) the personal financial burden for health services is considered as being too high; about equally high (n = 159) is the proportion of persons who criticize the communication with health professionals, especially hospitals and doctors' surgeries. Every third who mentions a medical practice uses terms such as "uncertainty" and "anxiety". It is conspicuous that men more often than women write that they feel unfairly treated in the health system (62% vs. 38%, p < 0.05).

**Conclusion:**

Predominantly older persons seek the assistance of the federal government commissioner for patient issues. Considerable uncertainty and anxiety with respect to services and charges within the system of the German health insurances become evident. It is not possible from the data to draw conclusions concerning the impact of the commissioner's work on the health system. Nor do we gain any knowledge about the usefulness of the service for the individual. Therefore, evaluation of the political impact and the user satisfaction should follow.

## Background

Improving the position of patients within the health system is a vital aspiration in many countries with comparably high health care standards [1-6]. These endeavours are based on the well-founded – but empirically not sufficiently verified – assumption that participation of well informed patients and their representatives in decision-making processes would improve the different levels of health care [7].

In Germany, political efforts for a user-oriented approach are still quite new. Although patients' interests are already represented by numerous institutions with different focus [8], a legal regulation for the institutionalisation of patients' issues came about for the first time with the health reform in the year 2000: 30 nationwide model projects for independent patient consultation and user information were subsidized by leading associations of the health insurances (according §65b code of social law V.) [3]. The most important tasks of these facilities are to provide neutral consultation which is independent of particular interests of service providers, cost carriers, industry, and politics.

The position of patients was further improved when the health reform came into force in 2004: a commissioner of the federal government was introduced to look after patients' issues [9-11]. The tasks of the patient commissioner are defined in the code of social law as follows:

*"The federal government appoints a commissioner for patients' issues. [...] The task of the commissioner is to ensure that the issues of patients, particularly with respect to their rights on extensive and independent consultation and objective information by service providers, cost units and authorities in the health care sector and regarding participation in questions of ensuring medical care are considered. [...] For the realization of this task [...] the federal ministries offer the commissioner participation in all legal, regulatory or other important initiatives as far as rights and protection of patients are concerned or touched upon [...]".*[11]

From the beginning, this office was held by Helga Kühn-Mengel; she is member of the Bundestag for the Social Democratic Party (SPD). Therefore, the services offered by the patient commissioner cannot be considered as independent in the sense of the above described definition: she is dependent on politics as she is appointed by the respective federal government and takes part in political work. Her office with its multi-professional staff is financed by taxes.

According to the recent experiences of the patient commissioner and her team, the processing of letters and e-mails received from citizens is their principal activity apart from public relations work. The knowledge gained from the inquiries finds its way into the internal and external parliamentary work. For example: when claims about the costs for certain freely available medication increased (over-the-counter drugs), the patient commissioner explicitly informed the federal joint committee on this and thus let the committee members "take the pulse of the people concerned". The federal joint committee (G-BA) is a body for the self-administration of doctors, health insurances and hospitals; the body decides which medical outpatient or inpatient services are adequate, purposeful and cost effective and therefore should be a part of the service catalogue of the health insurances [12]. The patient commissioner has no vote in the federal joint committee [11].

By taking a look some other countries it becomes evident that the institutions providing patients' support operate very differently under varying general frameworks [10]. In Great Britain, for example, ombudspersons work as representatives of the health authority, apart from this there are voluntary community health councils (CHCs) on a local level [8]. New Zealand has an ombudsman (health and disability commissioner) in a central, politically independent role, who has rather comprehensive possibilities to investigate individual cases and to provide recommendations [6,13]. Ombudspersons work on local, partly on nationwide levels, as also for example in Finland, Sweden, Norway, the Netherlands and Austria; in view of their structures, responsibilities, authorities and tasks they clearly differ from each other [8,10].

The patient commissioner of the federal government in Germany represents a unique institution of patients' support. In contrast to the ombudspersons in Great Britain and New Zealand, for example, the German patient commissioner usually does not follow up patients complaints in individual cases. Her focal points are provision of user friendly information on legal foundations and contact details on authorized institutions such as mediation services of the medical council, the consumer counselling centres and counselling facilites of the sickness funds.

For the first time, this study investigates which groups of persons approach the patient commissioner and which are the most important topics.

## Methods

The letters and e-mails of patients and citizens processed by the commissioner and her team during the timeframe January 01 – July 15, 2005, were investigated (n = 2580). From these documents, a 33% sampling was taken (n = 850), i. e. every third conclusively processed inquiry in the sequence of the chronological archiving was considered. Using combined qualitative (inductive category development, grounded theory method [14,15]) and quantitative procedures, the material was thematically encoded in the following way:

▪ compilation of socio demographic variables of the inquirers with domicile, postcode, gender, age and position (personally concerned/partner, friend of a patient) as well as the type of inquiry (letter – hand written as the case may be – and e-mail) and date of inquiry;

▪ a characterization of the inquiry was carried out whereby the contents and the manner (nature) were firstly described in a free text form;

▪ categories were derived from the extracted material using the inductive category development [14], whereby the material was devided into five main categories and partly into sub-categories (category 1);

▪ the allocation of the inquiries to the categories was done with the possibility of multiple nominations.

The coding process was primarily carried out by the principal investigator (NSCH) with support from a medical data assistant student. The core study team (NSCH, MLD, GS) discussed the category development during multiple workshops.

For evaluation purposes the statistic programme SPSS (Version SPSS 13.0 for Windows) was used. The results are presented in descriptive form and investigated especially with respect to sex and age-specific differences. Furthermore, the interdependences between the categorized criteria are analysed. The statistical level of significance was set at a probability value of 5% (Chi-square: p < 0.05).

## Results

### Who contacts the patient commissioner?

There is a tendency that older citizens rather then younger ones (average age 63 years, min.-max: 12 to 94 years) appeal to the patient commissioner. Details regarding age groups and sex distribution are shown in table 1.

**Table 1:** Investigated population

	n	%
**sex (n = 806)**		

men	410	50.9
women	396	49.1

age (n = 186) *		

80 years and older	22	11.8
70–79	44	23.7
60–69	63	33.9
50–59	25	13.4
40–49	17	9.1
30–39	10	5.4
20–29	1	0.5
19 years and younger	4	2.2

The absolute age of the inquirers could only be determined in 186 cases. In 301 other cases the inquirers could be assigned with high probability to the group of the older population, for example due to phrasings such as "I am a pensioner" or "I am an old woman".

The share of inquiries allocated to the 16 German federal states tends to be in proportion to the number of inhabitants. Greater deviations only occur concerning the citizens of the federal capital of Berlin; they represent 10.8% of the people writing to the patient commissioner, but only make up 4.1% of the population in Germany.

Most of the inquiries received are letters (n = 696 vs. e-mails n = 154). Almost one quarter of the letters are handwritten (n = 163). Predominantly women (24.7% vs. 14.6% of men) and older citizens (24.8% vs. 12.3% of the inquirers younger than 60 years) send in handwritten letters.

In most cases the person inquiring is the person concerned (78.1%; n = 664). 14.5% of the letters and e-mails are written by a family member or friend of the person concerned, 2.9% are written by a service provider (for example home care, doctor, pharmaceutical company), and 1.3% by a patient representation entity (self help group, patient organization). Table 2 and 3 show the allocation of the inquiries to the different categories respectively subcategories. The main results are described below.

**Table 2:** Structure of categories and allocation of the inquiries (total: n = 850) to the categories (multiple nominations)

**Category 1: Main topics**	n
Services of the legal health insurances	643
Diseases	432
Alternative medicine	61
Home care	49
Prevention/health promotion	38
Dentistry	36
New diagnostic/therapeutic procedures	30
Rehabilitation	28
Treatment errors	28
Pension	19

**Category 2: Institutions and services**	

Cost carriers	285
Doctors' offices	191
Politics	163
Hospitals	49

**Category 3: Perception of care delivery**	

Style, interaction and communication	159
Procedures of health care delivery	95

**Category 4: Attitudes and emotions**	

Personal financial burden	168
Anxiety and uncertainty	156
Injustice	120

**Category 5: Expectations and motivations**	

Complaints and resentment	609
Request for information	178
Request for personal support	89

**Table 3:** Sub-categories of category 1 (main topics) and allocation of the inquiries to the categories (multiple nominations)

**Services of the legal health insurances**	n
Medication (general)	224
Over-the-counter drugs	139
Medical adjuvants	94
Regulations for chronic diseases	81
Practice charge	55
Individual health services	35
Travel expense	33

**Diseases**	

Musculoskeletal system	117
Pain	106
Cardiovascular diseases	68
Cancer	63
Incontinence	31
Skin	26
Gynecological problems	24
Dementia	21
Eyes	21
Urology	21
Diabetes	16
Mental illness	14

### Main topics

Most of the inquiries concern the services of the health insurances (75.6%; n = 643) with a focus on medication (26.4%; n = 224). Freely available medication (OTC-medication) is expressly referred to in 16.4% of the cases (n = 139), increasingly in old age (10.5% of the <60-year olds vs. 28.8% of the 70-year olds and older; p < 0.05).

In half of the cases (50.8%; n = 432) the focus is on certain types of diseases. Mainly chronic diseases of the musculoskeletal system and pain are described, in fact, definitely more often by women than men (musculoskeletal system: 21% vs. 8%; p < 0.05, and pain: 16.4% vs. 8.8%; p < 0.05). There are statistically meaningful age specific differences with cardiovascular diseases which, as expected, are addressed significantly more frequently with increasing age (1.8% of the under 60-year-olds vs. 14.3% of the 60–69-year-olds vs. 18.2% of the ≥70-year-olds; p < 0.05).

In contrast, other thematic areas like travel expenses, practice charge as well as the so called individual health care services (i.e. services that have to be personally paid for and are not covered by the health insurances) are less frequently mentioned (less than 7% in each case).

Table 4 presents sex specific and age specific differences of the main topics.

**Table 4:** Main topics of the inquiries (multiple nominations) with gender specific and age specific differences *

**Main topics**	n	%	%	%	%	%	%
	total	total	men	women	<60 years	60–69 years	>70 years

Services of the health insurances	643	75.7	*71.5	*80.1	75.4	84.1	84.8
Diseases	432	51.6	*45.9	*57.6	57.9	54.0	60.6
Alternative medicine	61	7.6	*4.9	*10.4	12.3	7.9	4.5
Home care	49	6.1	5.1	7.1	**3.5	**4.8	**18.2
Prevention/health promotion	38	4.7	4.6	4.8	1.8	1.6	9.1
Dentistry	36	4.5	4.1	4.8	7.0	4.8	1.5
New diagnostic/therapeutic procedures	30	3.7	3.4	4.0	8.8	3.2	4.5
Rehabilitation	28	3.5	3.4	3.5	3.5	***7.9	***0.0
Treatment errors	28	3.5	3.2	3.8	7.0	***0.0	***6.1
Pension	19	2.4	*3.4	*1.3	1.8	1.6	6.1

* Statistically significant values in bold face print: Chi-square: p < 0.05 for *men vs. women, **<60–69 years old vs. ≥70 years old, ***60–69 years old vs. ≥70 years old.

### Style, interaction and communication

8.7% (n = 159) of the inquirers criticize style, interaction and communication of physicians, carers, hospital staff and other professionals. Looking at the persons only who mention hospitals, more than half explicitly mention this issue (57.1% vs. 16.4% of those who do not mention hospitals; p < 0.05). Regarding the persons referring to a doctor's office, it is 41.9% (vs. 12.0% of those referring not to a doctor's office; p < 0.05).

Over 70-year-olds criticize the communication issues remarkably often in connection with a doctor's office (59.1% vs. 9.3% of the <60-year olds; p < 0.05) and hospitals (55.6% vs. 15% of the <60-year-old; p < 0.05). Furthermore, 53.6% of the patients who suspect treatment errors, criticize communication of health professionals (vs. 17.5% of those who do not suspect treatment errors; p < 0.05).

### Anxiety, uncertainty, and feeling of injustice

8.4% (n = 156) of the inquirers explicitly refer to terms like "anxiety" and "uncertainty" (25.3% women vs. 12.2% men; p < 0.05). Every third person who mentions a doctor's office uses the terms "anxiety" or "uncertainty". This mainly applies to the older citizens: 40.9% of the inquirers 60-year old and older who mention doctors' offices write about anxiety and uncertainty (vs. 16.8% of those who do not mention doctors' offices; p < 0.05).

14.1% (n = 120) use the terms "injustice" and "disadvantage", whereby men are clearly predominant (62.4% vs. 37.6% of women; p < 0.05). Especially older people associate politics with "injustice" and "disadvantage" (47.1% of the 60-year-olds and older vs. 9.8% of the under 60-year-olds).

### Personal financial burden

In every fifth inquiry (19.8%; n = 168) it is expressed that the personal financial burden for health services is perceived as being too high; predominantly persons who refer to politics hold this opinion (25.8% vs. 18.3% of the persons who do not refer to politics; p < 0.05). This connection is significant with the over 60-year-olds and the over 70-year-olds, but not with the younger ones.

## Discussion

This first analysis of the German patient commissioner took place at a time of considerable upheaval in health policy. The effects of the renewed health reform at the beginning of the year 2004 received great public attention and had an relevant impact on the individual, for example the introduction of a practice charge for consulting a doctor's office. The relevance of the results for strengthened political focus on patient orientation is high.

Nationally as internationally there is still much room for improvement in case of enhanced data compilation on the patients' perspective, whereby Germany has to catch up particularly in comparison with countries like, for example, Great Britain and the Netherlands [3,16]. This study contributes to this, even if methodical weaknesses have to be considered.

### Study limitations

One weakness is the lacking feedback with the inquirers. The data were taken from the original written inquiries, without putting questions regarding understanding. An investigator bias is possible, as particularly the "soft" categories were subject to the personal evaluation of the principal investigator with support from a medical data assistant student. However, the principal investigator has a dual qualification in general medicine and public health and operated independently from the patient commissioner and other stakeholders.

The results are representative for the concerns of the citizens when they write to the patient commissioner, however, they are not representative for the concerns of German citizens. Furthermore, the results do not represent objective statements of the actual situation of health provision; they reflect subjective perceptions and needs of the inquirers.

The different structures and scope of activities of other national and international institutions acting for patients' interests make a comparison of the results difficult. However, comparisons are at least in some points meaningful, especially with the national model projects for an independent patient consultation service. They are the only services in Germany that have been comprehensively evaluated so far.

### Utilization

Men and women take up the service of the patient commissioner in equal measure. This clearly differs from the national model projects, which are mainly used by women (>60%) [3]. The age of the enquirers is another important difference: the patients' commissioner is contacted by older population groups than the model projects (average age of the enquirers 63 years vs. 47 years [3]). The particularly strong utilization of the commissioner by the older population becomes also obvious if compared to the age structure of the German population; scarcely 19% of the citizens are 65 years and older [17].

An explicable reason is the significant increase in morbidity from the fifth life decade. Similar to the most developed countries, the exposure to chronic widespread diseases of the elderly such as cardiovascular and musculoskeletal diseases is the major challenge for the German health care system [18]. The patients' quality of life can be strongly impaired by pain, disability and reduced capability in the daily routine, and the available health care services in Germany do not sufficiently satisfy the patients' demands [19,20]. Therefore, it is not surprising that older people with chronic diseases are the major users of the patient commissioner's service. All health systems with predominant curative orientation are confronted with the need to improve the consideration of the issues of the increasing group of older and chronically ill patients [21].

Concerning pain therapy, the investigators get the impression that some inquiries could have been written with the cooperation of physicians as, since certain formulations come up again and again for example. It is possible, that some physicians try to express their resentment about the fee distribution [22] in this way. It can only be speculated whether the anxiety and uncertainty in many inquiries to the patient commissioner can be attributed to an exploitation of the patients through doctors.

It is noticable that the citizens of Berlin have disproportionately more inquiries compared to the citizens of other federal states. They may have better knowledge of the available services and a lower threshold to overcome for contacting an institution of the federal government than people who live in peripheral regions.

In principle, it is very important to identify socioeconomic and ethnical barriers on the part of the users, but our material is not suitable to draw conclusions concerning these issues. To give an example, we know from studies in New Zealand that patients from more deprived socioeconomic backgrounds are less likely to complain to the health commissioner than patients from more privileged backgrounds [23].

### Financial burden

The "feeling" of financial overburdening of many people is an important message, confirming the trend shown in a telephone survey by the Bertelsmann Foundation: The Germans clearly feel their increased financial share in health services, after the last health reform came into force [24]. Independent from an objective judgement of the individual financial burden, the excessive demand *felt *by many citizens should be considered very seriously in health and social policy.

An increasing financial burden with German patients can be attributed mainly to the introduction of the practice charge, increased surcharges to or respective self-payments of over-the-counter drugs as well as to the so-called individual health services. The latter are methods or certain preventative medical check-ups, which are not part of the defined spectrum of services of the health insurances but increasingly offered in doctors' offices. Interesting in this context are the results of recent representative surveys among German physicians: Not every one proves to be sufficiently informed about the valid catalogue of services of the health insurances [25], which admits the assumption that some services – knowingly or unknowingly – are withheld from the patients or have been privately billed although they could have been charged to the account of the health insurances.

### Treatment errors

Treatment errors are not at the centre of the inquiries to the patient commissioner. In such cases patients seem to turn to the regional service points, for example model projects, mediation service of the medical council, the consumer counselling centres or counselling facilities of the sickness funds [3]. If patients address treatment errors to the patient commissioner they often criticize the communication style of doctors and other health professionals. This underlines the outstanding importance of a well functioning communication between patients and the people who treat them [2,5,6,26,27].

## Conclusion

Predominantly older persons seek the assistance of the federal government commissioner for patient issues. Considerable uncertainty and anxiety with respect to services and charges within the system of the German health insurances become evident. It is not possible from the data to draw conclusions concerning the impact of the commissioner's work on the health system, nor do we gain knowledge about the usefulness of the service for the individual.

Therefore, evaluation of the political impact and the user satisfaction should follow.

## Competing interests

The author(s) declare that they have no competing interests.

## Authors' contributions

NSCH conceived the study, performed the analyses and drafted the manuscript. MLD helped to conceive the study, to interpret the data, and to draft the manuscript. GS assisted in performing the analyses. FWS helped to conceive the study and to interpret the data. All authors read and approved the final manuscript.

## Pre-publication history

The pre-publication history for this paper can be accessed here:


